# Severe but Not Moderate Vitamin B12 Deficiency Impairs Lipid Profile, Induces Adiposity, and Leads to Adverse Gestational Outcome in Female C57BL/6 Mice

**DOI:** 10.3389/fnut.2016.00001

**Published:** 2016-01-22

**Authors:** Shampa Ghosh, Jitendra Kumar Sinha, Uday Kumar Putcha, Manchala Raghunath

**Affiliations:** ^1^Endocrinology and Metabolism Division, National Institute of Nutrition, Indian Council of Medical Research, Hyderabad, India; ^2^Pathology Division, National Institute of Nutrition, Indian Council of Medical Research, Hyderabad, India

**Keywords:** adipocytokines, altered lipid profile, homocysteine, inflammation, oxidative stress, vitamin B12 deficiency, mouse model, antioxidant activity

## Abstract

Vitamin B12 deficiency is widely prevalent in women of childbearing age, especially in developing countries. In the present study, through dietary restriction, we have established mouse models of severe and moderate vitamin B12 deficiencies to elucidate the impact on body composition, biochemical parameters, and reproductive performance. Female weanling C57BL/6 mice were fed for 4 weeks: (a) control AIN-76A diet, (b) vitamin B12-restricted AIN-76A diet with pectin as dietary fiber (severe deficiency group, as pectin inhibits vitamin B12 absorption), or (c) vitamin B12-restricted AIN-76A diet with cellulose as dietary fiber (moderate deficiency group as cellulose does not interfere with vitamin B12 absorption). After confirming deficiency, the mice were mated with male colony mice and maintained on their respective diets throughout pregnancy, lactation, and thereafter till 12 weeks. Severe vitamin B12 deficiency increased body fat% significantly, induced adiposity and altered lipid profile. Pregnant dams of both the deficient groups developed anemia. Severe vitamin B12 deficiency decreased the percentage of conception and litter size, pups were small-for-gestational-age and had significantly lower body weight at birth as well as weaning. Most of the offspring born to severely deficient dams died within 24 h of birth. Stress markers and adipocytokines were elevated in severe deficiency with concomitant decrease in antioxidant defense. The results show that severe but not moderate vitamin B12 restriction had profound impact on the physiology of C57BL/6 mice. Oxidative and corticosteroid stress, inflammation and poor antioxidant defense seem to be the probable underlying mechanisms mediating the deleterious effects.

## Introduction

Undernutrition is a major crisis in the developing world, particularly among women and children (1). The long-term consequences of nutritional imbalance in early life include chronic diseases in adulthood (2). Maternal undernutrition is an important determinant of poor gestational outcome, such as smaller size of infant at birth; small-for-gestational-age is associated with physiological and psychological disturbances in adulthood (3). UNICEF reports that 16% of newborns in 2013 had low birth weight (LBW), and its incidence was the highest in South Asia where one out of four newborns had LBW (http://data.unicef.org/nutrition/low-birthweight.html accessed on November 14, 2015). LBW is considered a strong risk factor for metabolic syndrome (4).

Micronutrient deficiencies during crucial phases of life have long-lasting repercussions on health (5). Vitamin B12 deficiency is a significant, global public health problem (6, 7). In developing countries like India, vitamin B12 deficiency is common among women and children (8). Vitamin B12 deficiency elevates the levels of plasma homocysteine and methylmalonic acid (9, 10) which in turn predispose the individuals to cognitive dysfunction, occlusive vascular disease, various malformations, and adverse pregnancy outcomes (11–14). Vitamin B12 deficiency is an independent risk factor leading to neural tube defects (15) and neurological complications (16–18). It also increases the risk of developing preeclampsia, intrauterine growth retardation, preterm labor, and recurrent abortions (19–22).

Considering that (i) nutritional status is an important predictor of health outcomes in the mother and offspring, (ii) different levels of vitamin B12 deficiency is rampant among women of reproductive age, and (iii) appropriate experimental animal models are unavailable, it was pertinent to study the effects of chronic vitamin B12 deficiency in an animal model from weaning to postreproductive age through pregnancy and lactation. In this study, we have evaluated the differential effects of severe and moderate vitamin B12 deficiency on body composition, adiposity, hematological parameters, lipid profile, blood glucose, and insulin in a C57BL/6 mouse model right from weaning to postreproductive age. We have also delineated oxidative stress, inflammation, and poor antioxidant defense as the probable mechanisms associated with the adverse effects of vitamin B12 deficiency.

## Materials and Methods

### Animal Maintenance and Feeding

The study was approved by the Institutional Animal Ethics Committee (National Institute of Nutrition, Hyderabad) and the Committee for the Purpose of Control and Supervision of Experiments on Animals (CPCSEA) (Regd. No. 154/RO/c/1999/CPCSEA). All the experiments were carried out as per the norms in accordance with the National Institute of Health Guide for the Care and Use of Laboratory Animals (NIH Publications No. 80-23) revised in 1996. The study was carefully planned to minimize the number of mice used and their suffering.

Female weanling C57BL/6 mice (*n* = 30) were obtained from National Center for Laboratory Animal Sciences, Hyderabad. The mice were housed in polypropylene cages and maintained at 22°C ± 2, under standard lighting conditions (12-h light/dark cycle). They were randomly divided into three groups and fed *ad libitum*, either AIN-76A control diet (Group 1, *n* = 10, designated as C) or the same diet deficient in vitamin B12 with pectin as the source of fiber (Group 2, *n* = 10, designated as B12R^+^) or without pectin (with cellulose as fiber source) (Group 3, *n* = 10, designated as B12R^−^) (Research Diets Inc., New Brunswick, NJ, USA). The content of vitamin B12 in the restricted diets was 62.5% lesser than the control diet (0.006 vs. 0.016 mg/kg diet); vitamin B12-restricted diet with pectin (B12R^+^) contained 50 g pectin/kg diet, because it has been shown earlier that pectin binds the intrinsic factor in the intestine and makes vitamin B12 less bioavailable (23). The control diet as well as vitamin B12-restricted diet without pectin (B12R^−^) contained 50 g cellulose/kg diet as the fiber source instead of pectin. The mice also had *ad libitum* access to deionized water. Food intake was recorded daily and body weights every week.

After 4 weeks of feeding, concentrations of vitamin B12, folate, homocysteine, glucose, insulin, cholesterol, triglycerides, high-density lipoprotein (HDL), and various adipocytokines were assessed in the plasma. Hematological parameters [RBC count, hematocrit%, hemoglobin, mean corpuscular hemoglobin concentration, and mean corpuscular volume (MCV)] were also evaluated. After confirming the deficiency, the mice were mated with control males (two females to one male). Successful conception was computed based on the ratio of the number of mice that were kept for breeding to the number of mice that conceived and was expressed as percentage. The female mice continued on their respective diets throughout pregnancy, lactation, and thereafter. Body weights of the mice were recorded before mating and weight gain during pregnancy was also monitored. Reproductive performance of the mice in different groups was closely monitored and recorded. Following the periods of gestation and lactation, after 12 weeks of continued feeding on their respective diets, vitamin B12, homocysteine, and various biochemical parameters were determined in plasma and body composition was also assessed by dual energy X-ray absorptiometry (DEXA). Further, oxidative stress markers and antioxidant enzyme activity were determined in liver samples as described below.

### Body Composition Analysis by Dual Energy X-Ray Absorptiometry

The body composition of mice from each group was monitored after 4 weeks (before mating) and 12 weeks (after weaning of pups) of feeding their respective diets by using DEXA (Discovery, Hologic, Bedford, MA, USA). Adiposity index (AI), an index of visceral adiposity, was calculated following the method of Taylor and Philips (24). Briefly, three major fat pads (retroperitoneal, mesenteric, and gonadal) were quickly dissected at the end of 12 weeks of feeding and their wet weights were recorded.

$$\begin{array}{c} \text{AI}=(\text{Sum of wet weights of retroperitoneal, mesenteric, }\\ \text{and gonadal fat deposits}/\text{Body weight})\times \text{1}00 \end{array}$$

### Biochemical Parameters

After overnight fasting, blood was collected from the supraorbital sinus of mice at the end of 4 and 12 weeks of feeding the respective diets. Fasting blood glucose was assessed from a drop of blood using a glucometer (Accu-Chek, Roche Diagnostics, USA). From the remaining blood sample, plasma was separated and stored at −80°C until further use. Plasma vitamin B12 and folate were determined on Immulite 2000 analyser using kit (Siemens Medical Solutions Diagnostics, Los Angeles, CA, USA) according to the manufacturer’s instructions. Plasma homocysteine concentrations were analyzed by high performance liquid chromatography (25).

Total cholesterol, triglycerides, and HDL cholesterol were measured in plasma using enzymatic assay kits (Biosystems, Barcelona, Spain) according to the manufacturer’s instructions.

Insulin and adipocytokines [leptin, tumor necrosis factor alpha (TNF-α), monocyte chemoattractant protein-1 (MCP-1), and interleukin-6 (IL-6)] were determined in fasting plasma using MILLIPLEX MAP Mouse Serum Adipokine Panel (EMD Millipore, Darmstadt, Germany) on a Bio-Plex 200 platform (Bio-Rad, CA, USA) following the manufacturer’s instructions.

### Hematological Parameters

Blood drawn from the orbital sinus of mice was collected in ethylenediaminetetraacetic acid (EDTA)-coated tubes to assess hematological parameters for the evaluation of anemia. For the analysis of complete blood picture, 50 μL of whole blood sample was fed into the Advia 120 Hematology System (Global Siemens Healthcare, Erlangen, Germany).

### Evaluation of Markers of Stress

Quantitative estimation of plasma cortisol was done by solid-phase, competitive chemiluminescent enzyme immunoassay on Immulite1000 platform using kit (Siemens Medical Solutions Diagnostics, CA, USA) as per the manufacturer’s instructions.

Markers of oxidative stress and antioxidant defense were determined in the liver homogenate. Lipid peroxidation was measured by quantifying malondialdehyde (MDA) spectrophotometrically at 532 nm (26) and protein carbonyls were estimated at 370 nm using 2,4-dinitrophenylhydrazine (27). Superoxide dismutase (SOD) was estimated according to the procedure of Marklund and Marklund (28) in the cytosolic fraction (100,000 × *g* supernatant) of liver tissue. One unit of SOD activity is taken as the amount of the enzyme that inhibits the rate of auto-oxidation of pyrogallol by 50% per minute and is expressed as units/min/mg protein. The activity of catalase was determined according to the method of Aebi (29) by the reduction of hydrogen peroxide.

### Statistical Analysis

The results are presented as mean ± SEM and the data was analyzed using Graphpad Prism 5.0.1 software (Graphpad Software Inc., San Diego, CA, USA). Significance of difference among the three groups of mice for each parameter studied was analyzed by one-way analysis of variance (ANOVA) followed by Bonferroni’s *post hoc* test appropriately. Differences were considered significant if “*p*” was at least ≤0.05.

## Results

### Establishing a Mouse Model for Vitamin B12 Deficiency

After 4 weeks of feeding the respective diets to weanling female C57BL/6 mice, we measured the levels of vitamin B12 and homocysteine in their plasma. Concentration of vitamin B12 in the plasma of B12R^+^ and B12R^−^ mice was significantly lower than that of control mice. As expected, among the B12-restricted groups, B12R^+^ mice had lower plasma vitamin B12 concentrations than B12R^−^ mice, showing severe deficiency. On the other hand, plasma homocysteine levels were significantly higher in both the restricted groups as compared to control (Table 1). In line with their lowest plasma vitamin B12 levels, the plasma homocysteine levels were the highest in B12R^+^ mice. The levels of folate were comparable among all the three study groups. In line with available literature, dietary fiber pectin inhibited vitamin B12 absorption and led to severe deficiency in B12R^+^ mice. On the other hand, dietary fiber cellulose did not interfere with vitamin B12 absorption and the B12R^−^ mice developed moderate deficiency. Thus, 4 weeks of feeding vitamin B12-restricted AIN-76A diet with different dietary fibers pectin and cellulose to weanling C57BL/6 mice successfully established severe and moderate vitamin B12-deficient mouse models, respectively.

**Table 1:** **Plasma vitamin B12, folate, and homocysteine levels after 4 and 12 weeks of feeding**.

	Feeding duration (weeks)	C	B12R^+^	B12R^−^
Plasma vitamin B12 (pg/mL)	4	565 ± 12^a^	184 ± 11^b^	253 ± 11^c^
	12	406 ± 17.6^a^	138 ± 11.9^b^	208 ± 9.1^c^
Plasma folate (ng/mL)	4	26 ± 1.0	25.0 ± 1.1	27.0 ± 0.7
	12	26.6 ± 1.37	27.3 ± 0.98	26.8 ± 1.29
Plasma homocysteine	4	3.9 ± 0.56^a^	9.7 ± 0.55^b^	8.0 ± 0.57^b^
	12	4.2 ± 0.4^a^	10.5 ± 1.21^b^	8.7 ± 0.78^c^

*Values are mean ± SEM (*n* = 6)*.

*Values with different superscripts (a/b/c) are significantly different from one another at *p* < 0.05 by one-way ANOVA/Bonferroni’s *post hoc* test*.

### Effect of Vitamin B12 Restriction on Lipid Profile, Fasting Glucose, and Insulin Levels after 4 Weeks of Feeding

As seen in Table 2, after 4 weeks on vitamin B12 deficient diet, the severely deficient B12R^+^ mice showed significantly higher levels of plasma cholesterol than control mice. Similar observation was seen in case of triglycerides levels where B12R^+^ mice had significantly higher levels as compared to both the control as well as moderately deficient mice. Although HDL cholesterol, homeostatic model assessment of insulin resistance (HOMA-IR) index, fasting glucose as well as fasting insulin levels were increased in B12R^+^ mice, the differences were not significant (Table 2).

**Table 2:** **Lipid profile, fasting glucose, and insulin levels and HOMA IR after 4 and 12 weeks of feeding**.

	Feeding duration (weeks)	C	B12R^+^	B12R^−^
Cholesterol (mmol/L)	4	1.98 ± 0.074^a^	2.5 ± 0.16^b^	2.0 ± 0.13^ab^
	12	1.95 ± 0.047^a^	2.79 ± 0.209^b^	2.12 ± 0.139^a^
Triglycerides (mmol/L)	4	0.49 ± 0.025^a^	0.64 ± 0.035^b^	0.54 ± 0.033^a^
	12	0.49 ± 0.028^a^	0.71 ± 0.029^b^	0.55 ± 0.037^a^
HDL (mmol/L)	4	1.45 ± 0.071	1.55 ± 0.077	1.45 ± 0.096
	12	1.57 ± 0.742^a^	1.12 ± 0.068^b^	1.48 ± 0.115^a^
Fasting glucose (mmol/L)	4	4.7 ± 0.20	5.2 ± 0.17	5.0 ± 0.19
	12	5.55 ± 0.217	6.97 ± 0.409	6.05 ± 0.467
Fasting insulin (pmol/L)	4	155.0 ± 7.8	159.0 ± 8.8	159.0 ± 6.9
	12	117 ± 5.5	148 ± 10.1	134 ± 10.0
HOMA-IR (units)	4	4.5 ± 0.31	5.1 ± 0.27	4.9 ± 0.28
	12	4.03 ± 0.343	6.4 ± 0.778	5.14 ± 7.07

*Values are mean ± SEM (n = 6)*.

*Values with different superscripts (a/b) are significantly different from one another at *p* < 0.05 by one-way ANOVA/Bonferroni’s *post hoc* test*.

### Effect of Vitamin B12 Restriction on Body Composition after 4 Weeks of Feeding

**Figure 1:**
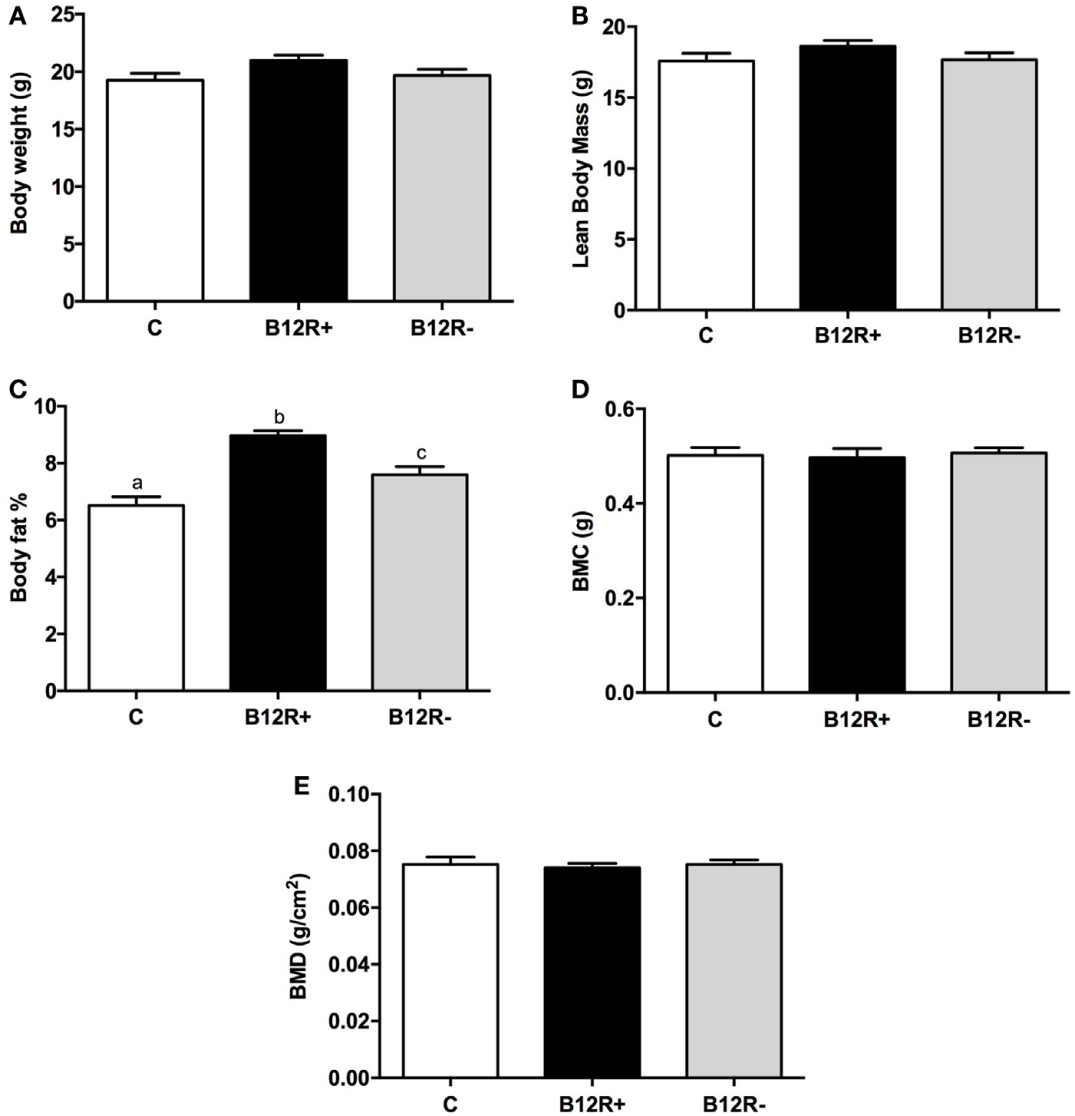
**Body composition analysis by DEXA after 4 weeks of feeding: (A) body weight, (B) lean body mass, (C) body fat%, (D) body mineral content, and (E) body mineral density**. Bars with different superscripts (a/b/c) are significantly different from one another at *p* < 0.05 by one-way ANOVA/Bonferroni’s *post hoc* test.

Body composition analysis of the mice using DEXA showed no significant changes in the bone mineral content (BMC), bone mineral density (BMD), and lean body mass (LBM) after 4 weeks of feeding the experimental diets (Figure 1). However, body fat percentage showed a significant rise in both the vitamin B12-deficient groups as compared to control mice. Among the two deficient groups, severely deficient B12R^+^ mice had significantly higher body fat percentage than moderately deficient B12R^−^ mice.

### Status of Plasma Adipocytokines after 4 Weeks of Feeding

Plasma TNF-α and IL-6 levels were significantly elevated in severely deficient B12R^+^ mice compared to controls, whereas other plasma adipocytokine levels were comparable among the groups after 4 weeks of feeding the respective diets (Table 3).

**Table 3:** **Plasma adipocytokine levels after 4 and 12 weeks of feeding**.

Adipocytokines	Feeding duration (weeks)	C	B12R^+^	B12R^−^
TNF-α (pg/mL)	4	117 ± 3.5^a^	143 ± 5.0^b^	131 ± 5.1^ab^
	12	119 ± 5.8^a^	150 ± 6.6^b^	134 ± 5.5^ab^
Leptin (ng/mL)	4	5.6 ± 0.21	6.1 ± 0.25	5.8 ± 0.25
	12	5.8 ± 0.39^a^	9.9 ± 0.68^b^	6.5 ± 0.36^a^
MCP-1 (pg/mL)	4	43.0 ± 1.18	46.9 ± 2.08	44.4 ± 1.95
	12	43.2 ± 2.47	49.5 ± 1.27	45.3 ± 3.02
IL-6 (pg/mL)	4	150 ± 3.2^a^	184 ± 8.0^b^	154 ± 4.5^a^
	12	151 ± 6.7^a^	185 ± 8.2^b^	176 ± 11.0^ab^

*Values are mean ± SEM (*n* = 6)*.

*Values with different superscripts (a/b) are significantly different from one another at *p* < 0.05 by one-way ANOVA/Bonferroni’s *post hoc* test*.

### Effect on Hematological Parameters

The hematological parameters, such as RBC count, hematocrit percentage, hemoglobin, mean corpuscular hemoglobin concentration, and MCV, showed a significant drop in the B12R^+^ mice as compared to controls. The hemoglobin was significantly lower in both the deficient groups, but there was no difference in MCVs of the different animal groups (Table 4).

**Table 4:** **Hematological parameters after 4 weeks of feeding (before breeding)**.

	C	B12R^+^	B12R^−^
RBC count (×10^6^/mm^3^)	10.8 ± 0.24^a^	9.45 ± 0.201^b^	9.61 ± 0.179^ab^
Hematocrit %	47.3 ± 1.31^a^	42.4 ± 1.00^b^	44.9 ± 0.75^ab^
Hemoglobin (g/dL)	15.0 ± 0.36^a^	9.95 ± 0.694^b^	12.2 ± 0.62^c^
Mean corpuscular hemoglobin concentration (g/dL)	31.8 ± 1.36^a^	23.6 ± 1.86^b^	27.3 ± 1.71^ab^
Mean corpuscular volume (μm^3^)	44.0 ± 1.36	44.5 ± 1.26	46.8 ± 1.22

*Values are mean ± SEM (*n* = 6)*.

*Values with different superscripts (a/b/c) are significantly different from one another at *p* < 0.05 by one-way ANOVA/Bonferroni’s *post hoc* test*.

### Effect of Vitamin B12 Deficiency on Gestational Outcome

Severely deficient B12R^+^ mice showed very poor gestational outcome (Table 5) as compared to both control and moderately deficient B12R^−^ mice. There was around 35% weight gain during pregnancy in the B12R^+^ mice as compared to approximately 51 and 47% in control and B12R^−^ mice, respectively. There was delayed conception in B12R^+^ mice and the percentage of successful conception in B12R^+^ was half of that observed in the control and B12R^−^ mice. The litter size was very low (i.e., 2) in case of severe deficiency as compared to 8 and 7 in control and B12R^−^ mice, respectively. The body weight and body length (nose to base of tail) at birth were significantly lower in pups of both the severe and moderately deficient dams clearly indicating that these pups were small for gestational age. A high percentage of pups born to B12R^+^ dams died during lactation period compared to both control and B12R^−^ dams. The body weight of pups from B12R^+^ mothers remained significantly lower even at weaning as compared to those born to control and B12R^−^ dams (Table 5).

**Table 5:** **Gestational outcome**.

	C	B12R^+^	B12R^−^
% of successful conception	100^a^	50^b^	100^a^
% Weight gain during pregnancy	51 ± 3.5^a^	34.5 ± 4.09^b^	46.8 ± 2.68^a^
Litter size	8.0 ± 0.37^a^	2.0 ± 0.76^b^	7.0 ± 0.32^a^
% deaths (of pups) during lactation	10.6 ± 3.61^a^	75.0 ± 14.40^b^	11.7 ± 3.45^a^
Birth weight of pups (g)	1.3 ± 0.06^a^	0.83 ± 0.029^b^	1.1 ± 0.04^c^
Body length of pups at birth (nose to base of tail) (cm)	3.8 ± 0.06^a^	2.6 ± 0.08^b^	3.2 ± 0.10^c^
Weaning weight of pups (g)	9.9 ± 0.31^a^	7.3 ± 0.40^b^	9.8 ± 0.36^a^

*Values are mean ± SEM (*n* = 10)*.

*Values with different superscripts (a/b/c) are significantly different from one another at *p* < 0.05 by one-way ANOVA/Bonferroni’s *post hoc* test*.

### Effect of Vitamin B12 Restriction on Plasma Vitamin B12 and Homocysteine Levels after 12 Weeks of Feeding

After 12 weeks of feeding different experimental diets, we observed significantly decreased levels of vitamin B12 in both B12R^+^ and B12R^−^ mice as compared to control mice (Table 1). The plasma homocysteine was also significantly high in both B12R^+^ and B12R^−^ as compared to the control mice. However, there were no changes in the plasma folate levels between the groups.

### Effect of Vitamin B12 Restriction on Body Composition after 12 Weeks of Feeding

**Figure 2:**
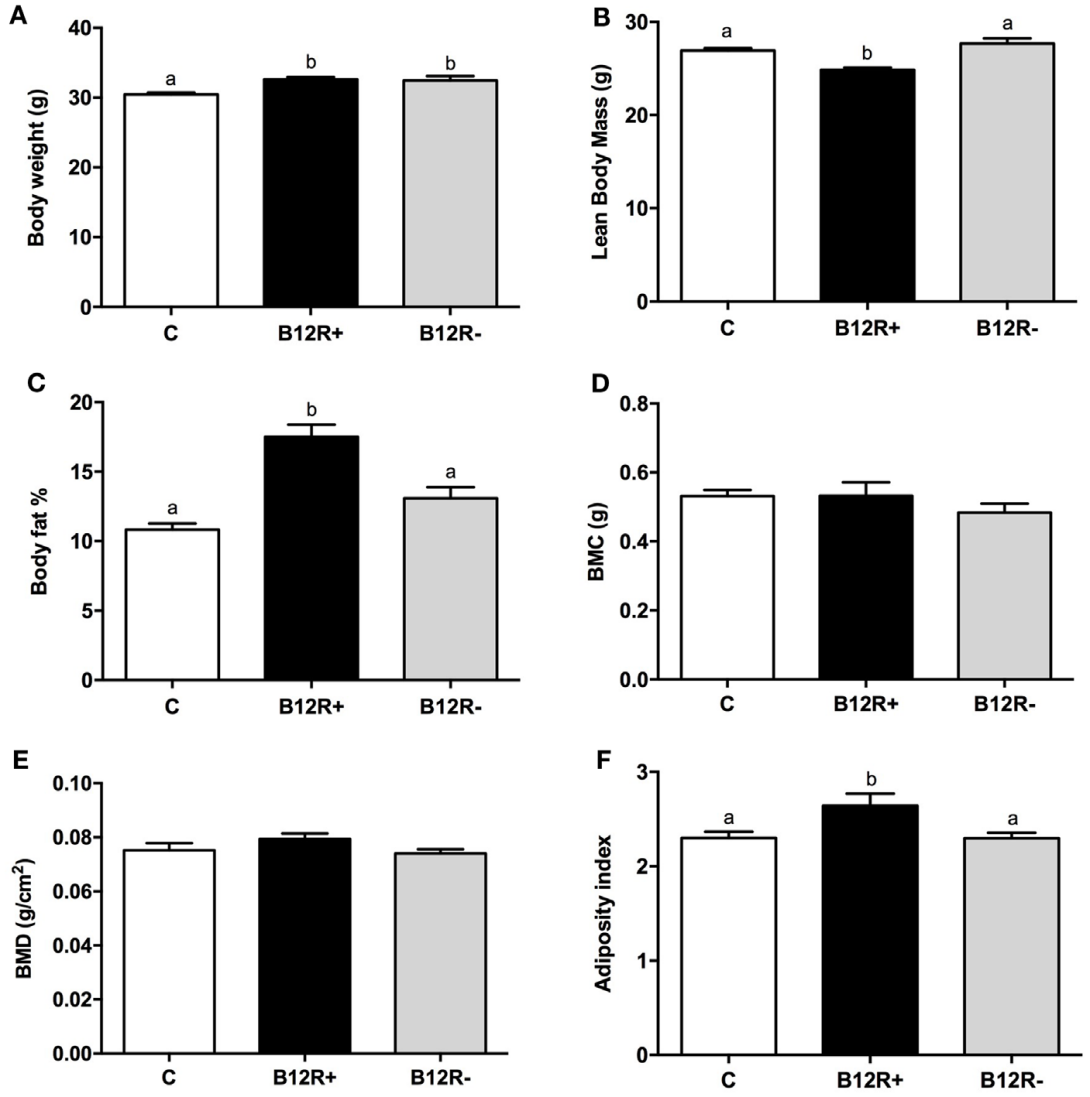
**Body composition analysis by DEXA after 12 weeks of feeding: (A) body weight, (B) lean body mass, (C) body fat%, (D) body mineral content, (E) body mineral density, and (F) adiposity index**. Bars with different superscripts (a/b/c) are significantly different from one another at *p* < 0.05 by one-way ANOVA/Bonferroni’s *post hoc* test.

As evident from Figure 2, 12 weeks of feeding experimental diets resulted in a significant increase in the body weights of both the deficient groups as compared to control group. Further, body composition analysis showed a significant increase in body fat percentage in severely deficient B12R^+^ mice but not in moderately deficient B12R^−^ mice. BMC and BMD remained unchanged even after 12 weeks of feeding-restricted diets. LBM was observed to be decreased and AI was increased in the severely deficient B12R^+^ mice as compared to control and moderately deficient B12R^−^ mice.

### Effect of Vitamin B12 Restriction on Lipid Profile, Fasting Glucose, and Insulin Levels after 12 Weeks of Feeding

Lipid profile was altered in the severely deficient mice after 12 weeks of feeding (Table 2). The severely deficient B12R^+^ mice had significantly higher levels of cholesterol and triglycerides than control and moderately deficient mice. On the other hand, HDL levels were significantly lower in B12R^+^ compared to both the control and B12R^−^ groups. After 12 weeks of feeding, the B12R^−^ mice did not show any significant changes in lipid profile parameters compared to controls. The levels of fasting glucose, fasting insulin, and HOMA-IR were higher in both the deficient groups but not statistically significant (Table 2).

### Status of Plasma Adipocytokines after 12 Weeks of Feeding

Only B12R^+^ mice showed significantly higher plasma TNF-α, leptin, and IL-6 levels compared to controls. Plasma TNF-α levels were comparable between the two deficient groups. However, B12R^+^ mice had significantly higher plasma leptin and IL-6 levels as compared to moderately deficient B12R^−^ mice. The plasma MCP-1 levels were comparable among the groups after 12 weeks of feeding the respective diets (Table 3).

### Status of Markers of Stress and Antioxidant Enzyme Activities after 12 Weeks of Feeding

**Figure 3:**
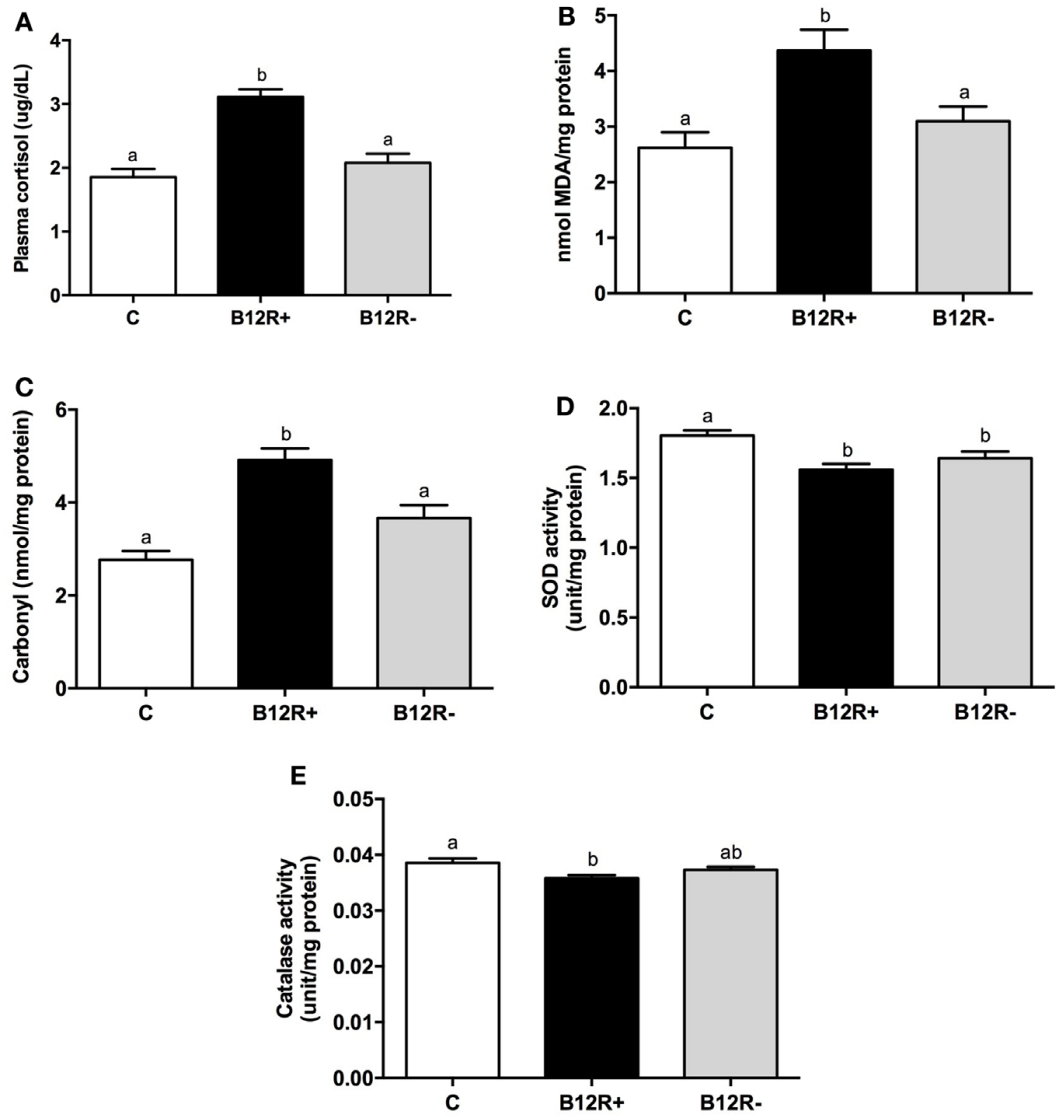
**Evaluation of markers of stress and antioxidant activity: (A) plasma cortisol level, (B) lipid peroxidation in liver, (C) protein carbonyl content in liver, (D) SOD activity in liver, and (E) catalase activity in liver**. Bars with different superscripts (a/b) are significantly different from one another at *p* < 0.05 by one-way ANOVA/Bonferroni’s *post hoc* test.

In the present study, both corticosteroid stress and oxidative stress were significantly increased in severely deficient B12R^+^ mice as compared to the control and B12R^−^ mice (Figure 3). After 12 weeks of feeding, plasma cortisol levels (Figure 3A) were significantly higher in B12R^+^ mice compared to control and B12R^−^ mice. In line with this finding, the levels of lipid peroxidation as well as protein carbonyls in liver (Figures 3B,C) were significantly higher in the B12R^+^ mice compared to control and B12R^−^ mice. Antioxidant enzyme activities (Figures 3D,E) were significantly reduced in the liver of B12R^+^ mice compared to the controls. Interestingly, SOD activity was significantly decreased in the liver of both the vitamin B12-restricted groups, whereas catalase activity was significantly decreased in the liver of B12R^+^ but not B12R^−^ mice as compared to the controls.

## Discussion

Vitamins and minerals (micronutrients) play a central role in cellular metabolism, maintenance, and growth throughout life and are helpful in prevention and/or cure of various disorders during the course of life including aging (30–32). Maternal undernutrition not only affects fetal growth and development but also increases the vulnerability to disorders such as diabetes and obesity significantly (21, 33, 34). Vitamin B12 is an important micronutrient essential for numerous vital processes in our body including normal functioning of the brain and nervous system and for the formation of blood. A regular dietary supply of vitamin B12 across the life course is essential due to its role as methyl donor and in vital functions of normal growth, development, and maintenance of various physiological functions (35). Deficiency of vitamin B12 during pregnancy is associated with neural tube defects, gestational diabetes, preeclampsia, recurrent pregnancy loss, fetal growth restriction, preterm labor, megaloblastic anemia, and neurological symptoms in the neonate (19, 20, 22, 36). To the best of our knowledge, there are no studies in a mouse model where the effects of vitamin B12 deficiency has been evaluated in terms of gestational outcome, lipid profile, levels of adipocytokines, hematological parameters, and oxidative stress levels. Considering that vitamin B12 deficiency is widespread among women from developing countries, in this study, we assessed the effects of chronic moderate and severe vitamin B12 deficiencies in female C57BL/6 mice. Pectin was added in B12R^+^ diet to create severe deficiency as it makes vitamin B12 less bioavailable and also promotes depletion of endogenous vitamin B12 due to its enterohepatic circulation (37).

Studies show that growth and development is significantly influenced by the maternal vitamin B12 status across pre- and postnatal periods (38). The embryo is primarily dependent on the maternal diet for all the required nutrients (39). The significant steps of development finally define the future health status as various cellular processes including differentiation and development depends upon *in utero* conditions (40). Therefore, poor nutritional status of mothers during periconceptional period results in preterm delivery, shorter birth-length, LBW (21), and likely neurodevelopmental disorders in the fetus (41). In the present study, we found significantly poor gestational outcome in the severely deficient B12R^+^ mice as compared to the controls, which was comparable to the moderately deficient B12R^−^ mice (Table 5). In previous studies from our laboratory, the reproductive performance in female Wistar rats was comparable among the vitamin-restricted and controls groups (42), which shows that C57BL/6 mice may be a more sensitive animal model for vitamin restriction experiments. Although (i) plasma vitamin B12 levels observed in the deficient C57BL/6 mice were in similar range as reported in vitamin B12-deficient humans (normal: >300 pg/mL; moderate deficiency: 201–300 pg/mL; and severe deficiency: <201 pg/mL) (43) and (ii) about 30,000 genes of the C57BL/6 mouse genome is known to have 99% direct counterparts in humans (44, 45), this animal model may not necessarily reiterate the exact features of vitamin B12 deficiency in humans (46, 47).

In the present study, we observed significantly increased body fat% in the B12R^+^ and B12R^−^ mice compared to the controls after 4 weeks feeding. Interestingly, after 12 weeks of feeding respective diets, B12R^+^ mice showed significantly increased body fat% compared to controls, whereas the increase in B12R^−^ mice was not significant. This is in line with the significant increase in body weight seen in both B12R^+^ and B12R^−^ mice. However, after 12 weeks of feeding respective diets, the LBM was decreased and AI increased significantly *albeit* in only the B12R^+^ mice compared to control and B12R^−^ mice. These results appear to suggest that the effects of a moderate deficiency of vitamin B12 may be masked due to unknown adaptive processes, wherein the body tries to maintain normalcy whereas severe deficiency could be precipitating significant, permanent changes in the phenotype and/or the adaptive processes are insufficient to handle the changes. Severe vitamin B12 deficiency from early life probably disturbs the metabolic pathways, thereby leading to obesity. Considering that (i) obesity is a known chronic low-grade inflammatory condition (48) and (ii) our results show proinflammatory adipocytokines, such as TNF-α and IL-6, are significantly increased as early as 4 weeks of feeding vitamin B12 deficient diet, suggesting that severe vitamin B12 deficiency in early life may increase the animal’s vulnerability to obesity. That it could be so, is corroborated by our finding that after 12 weeks of feeding, B12R^+^ mice had significantly higher levels of circulating proinflammatory adipocytokines, such as TNF-α, leptin, and IL-6 levels, compared to controls implicating sustained support to obesity, and this is in line with the increased body fat% of B12R^+^ mice at this time point.

Increased levels of proinflammatory cytokines, such as TNF-α and IL-6, have been associated with cases of depression (49) and as per a recent study, inhibition of TNF-α prevents cognitive decline and maintains hippocampal brain-derived neurotrophic factor (BDNF) levels (50). Therefore, with consistently increased levels of proinflammatory cytokines in severely deficient mice, the present study points to the probable involvement of vitamin B12 deficiency in reduced BDNF levels observed in brain and depression/cognitive deficits, which would be interesting to explore further.

In line with the observations in adipocytokine levels and body composition, we found the lipid profile to be deranged after 4 weeks of feeding, which was exacerbated after 12 weeks of feeding. The cholesterol and triglycerides levels were significantly higher in the B12R^+^ mice as compared to control and B12R^−^ mice both after 4 and 12 weeks, whereas the HDL levels were significantly higher only after 12 weeks of feeding. The levels of fasting glucose and fasting insulin were higher in the B12R^+^ mice, *albeit* not significant. Though not statistically significant, the higher HOMA-IR values, observed in the B12R^+^ mice as compared to controls, perhaps suggest their increased vulnerability toward insulin resistance.

Anemia has remained a public health problem in developed as well as developing countries (51, 52). As per the recent reports, 32 million pregnant women suffer from anemia globally, micronutrient deficiency being a potential cause (53). Vitamin B12 deficiency is known to be clinically associated with anemia (54). Though megaloblastic anemia is commonly linked to vitamin B12 deficiency, studies report normal MCV in vitamin B12 deficient patients (55, 56). Therefore, using MCV as an indicator to ascertain vitamin B12 deficiency may mask an actually serious deficiency condition. In our study as well, the MCV values were found to be comparable between the deficient and control mice, implicating that MCV might be an insensitive marker of vitamin B12 deficiency. However, we found significantly decreased values of RBC count, heamatocrit%, hemoglobin, and mean corpuscular hemoglobin concentration in the severely deficient mice compared to controls. The moderately deficient mice also had decreased values for the above parameters, but only hemoglobin content was significantly low. These observations clearly indicate the development of anemia due to the deficiency of vitamin B12 without macrocytosis.

Available literature suggests that increased levels of inflammatory cytokines, such as TNF-α, activate the hypothalamic-pituitary-adrenocortical (HPA) axis resulting in the release of cortisol (57). Therefore, we evaluated plasma cortisol levels in our mouse model. Significantly elevated cortisol levels in B12R^+^ mice as compared to controls and B12R^−^ mice appear to indicate dysregulation of HPA axis due to inflammation. Future studies exploring the regulation of HPA axis in detail in case of vitamin B12 deficiency would prove fruitful.

Many studies have reported that expression of genes related to inflammation and oxidative stress are significantly higher in the serum and placenta of obese women (58–61). Poor antioxidant defense and oxidative stress have been linked to adiposity in humans (62) and rodent models (63). Our observation of increased body fat% and proinflammatory cytokines in vitamin B12-deficient mice is in agreement with the foregone literature. It was therefore considered imperative to check the levels of oxidative stress and antioxidant damage in them. In agreement with literature, we found increased oxidative stress (levels of MDA and protein carbonyls) in severely deficient mice and diminished antioxidant defense (levels of SOD and catalase) in both the deficient groups as compared to controls. Therefore, it is evident from this study that both inflammation and oxidative stress may mediate the deleterious effects of vitamin B12 deficiency.

In summary, in this study, we have provided insights on the differential effects of moderate and severe vitamin B12 deficiencies in female C57BL/6 mice from early life, through gestation, lactation to postreproductive age in terms of body composition, reproductive performance, hematological status, lipid profile, and various other biochemical parameters. Also, our results implicate that inflammation and stress underlie the deleterious effects of vitamin B12 deficiency. Having established a mouse model of vitamin B12 deficiency successfully, our future studies would focus on the transgenerational changes of maternal vitamin B12 deficiency in F1 generation offspring and the involvement of epigenetic mechanisms.

## Author Contributions

The work presented here was conceived and designed by MR, SG, and JS. SG, JS, and UP carried out the experiments, analyzed the data, and interpreted the results. SG, JS, and MR wrote the paper. All the authors have contributed to and approved the manuscript.

## Conflict of Interest Statement

The authors declare that the research was conducted in the absence of any commercial or financial relationships that could be construed as a potential conflict of interest.

## Abbreviations

AI, adiposity index; AIN, American Institute of Nutrition; ANOVA, analysis of variance; B12R^−^, moderate vitamin B12 restriction; B12R^+^, severe vitamin B12 restriction; BDNF, brain-derived neurotrophic factor; BMC, bone mineral content; BMD, bone mineral density; C, control; DEXA, dual energy X-ray absorptiometry; EDTA, ethylenediaminetetraacetic acid; HDL, high-density lipoprotein; HOMA-IR, homeostatic model assessment of insulin resistance; HPA, hypothalamic–pituitary–adrenocortical; IL-6, interleukin-6; LBM, lean body mass; LBW, low birth weight; MCP-1, monocyte chemoattractant protein-1; MCV, mean corpuscular volume; MDA, malondialdehyde; SEM, standard error of the mean; SOD, superoxide dismutase; TNF-α, tumor necrosis factor alpha.
