# Supplementary material for: Severe but Not Moderate Vitamin B12 Deficiency Impairs Lipid Profile, Induces Adiposity, and Leads to Adverse Gestational Outcome in Female C57BL/6 Mice
Source: Front Nutr. 2016 Jan 22;3:1. doi: 10.3389/fnut.2016.00001 (PMC4722109; doi:10.3389/fnut.2016.00001)
Supplement: Supplementary file 1 [file Table_1.DOCX]

|  | Control (C) | Severe deficiency (B12R+) | Moderate deficiency  (B12R-) |
| --- | --- | --- | --- |
| Diet from weaning and continued throughout | AIN-76A  16ug vitamin B12/kg diet; Cellulose – dietary fiber | AIN-76A  6ug vitamin B12/kg diet; Pectin – dietary fiber | AIN-76A  6ug vitamin B12/kg diet; Cellulose – dietary fiber |
| Early life/ pre-reproductive age  (after 4 weeks of feeding) |  | 🡫 plasma vitamin B12  🡩 homocysteine  🡩 body fat %  🡩 cholesterol  🡩 triglycerides  🡩 TNF-α  🡩 IL-6  🡫 RBC Count  🡫 hematocrit %  🡫 hemoglobin  🡫 MCHC | 🡫 plasma vitamin B12  🡩 homocysteine  🡩 body fat %  -  -  -  -  -  -  🡫 hemoglobin  - |
| Reproductive age – pregnancy and lactation |  | 🡫 % of successful conception  🡩 weight gain during pregnancy  🡫 litter size  🡩 % of pup deaths during lactation  🡫 birth weight of pups  🡫 body length of pups  🡫 weaning weight of pups | -  -  -  -  🡫 birth weight of pups  🡫 body length of pups  - |
| Post reproductive age (after 12 weeks of feeding) |  | 🡫 plasma vitamin B12  🡩 homocysteine  🡩 body weight  🡫 lean body mass  🡩 body fat %  🡩 adiposity index  🡩 cholesterol  🡩 triglycerides  🡩 HDL  🡩 TNF-α  🡩 leptin  🡩 IL-6  🡩 plasma cortisol  🡩 lipid peroxidation  🡩 protein carbonyls  🡫 SOD activity  🡫 catalase activity | 🡫 plasma vitamin B12  🡩 homocysteine  🡩 body weight  -  -  -  -  -  -  -  -  -  -  -  -  🡫 SOD activity  - |

Supplementary Table 1: Effect of severe and moderate vitamin B12 deficiency in female C57BL/6 mice at early reproductive and post-reproductive ages. The significant increase or decrease of various parameters in the experimental groups is in comparison to control mice.
